# Diagnosis of neglected tropical diseases among patients with persistent digestive disorders (diarrhoea and/or abdominal pain ≥14 days): Pierrea multi-country, prospective, non-experimental case–control study

**DOI:** 10.1186/s12879-015-1074-x

**Published:** 2015-08-18

**Authors:** Katja Polman, Sören L. Becker, Emilie Alirol, Nisha K. Bhatta, Narayan R. Bhattarai, Emmanuel Bottieau, Martin W. Bratschi, Sakib Burza, Jean T. Coulibaly, Mama N. Doumbia, Ninon S. Horié, Jan Jacobs, Basudha Khanal, Aly Landouré, Yodi Mahendradhata, Filip Meheus, Pascal Mertens, Fransiska Meyanti, Elsa H. Murhandarwati, Eliézer K. N’Goran, Rosanna W. Peeling, Raffaella Ravinetto, Suman Rijal, Moussa Sacko, Rénion Saye, Pierre H. H. Schneeberger, Céline Schurmans, Kigbafori D. Silué, Jarir A. Thobari, Mamadou S. Traoré, Lisette van Lieshout, Harry van Loen, Kristien Verdonck, Lutz von Müller, Cédric P. Yansouni, Joel A. Yao, Patrick K. Yao, Peiling Yap, Marleen Boelaert, François Chappuis, Jürg Utzinger

**Affiliations:** Department of Biomedical Sciences, Institute of Tropical Medicine, Antwerp, Belgium; Department of Epidemiology and Public Health, Swiss Tropical and Public Health Institute, Basel, Switzerland; University of Basel, Basel, Switzerland; Institute of Medical Microbiology and Hygiene, Saarland University, Homburg/Saar, Germany; Division of Tropical and Humanitarian Medicine, Geneva University Hospitals, Geneva, Switzerland; Department of Paediatrics and Adolescent Medicine, B P Koirala Institute of Health Sciences, Dharan, Nepal; Department of Microbiology, B P Koirala Institute of Health Sciences, Dharan, Nepal; Department of Clinical Sciences, Institute of Tropical Medicine, Antwerp, Belgium; London School of Hygiene and Tropical Medicine, London, United Kingdom; Unité de Formation et de Recherche Biosciences, Université Félix Houphouët-Boigny, Abidjan, Côte d’Ivoire; Département Environnement et Santé, Centre Suisse de Recherches Scientifiques en Côte d’Ivoire, Abidjan, Côte d’Ivoire; Institut National de Recherche en Santé Publique, Bamako, Mali; Centre for Tropical Medicine, Faculty of Medicine, Gadjah Mada University, Yogyakarta, Indonesia; University of Cape Town, Cape Town, South Africa; Coris BioConcept, Gembloux, Belgium; Department of Pharmaceutical and Pharmacological Sciences, KU Leuven, Leuven, Belgium; Department of Internal Medicine, B P Koirala Institute of Health Sciences, Dharan, Nepal; Department of Epidemiology and Molecular Diagnostics, Agroscope Changins-Wädenswil ACW, Wädenswil, Switzerland; Department of Virology, Spiez Laboratory, Federal Office for Civil Protection, Spiez, Switzerland; Department of Parasitology, Leiden University Medical Center, Leiden, The Netherlands; Department of Public Health, Institute of Tropical Medicine, Antwerp, Belgium; Divisions of Infectious Diseases and Medical Microbiology, J.D. MacLean Centre for Tropical Diseases, McGill University Health Centre, Montreal, Canada

**Keywords:** Bacteria, Diagnosis-treatment algorithm, Helminths, Intestinal protozoa, Neglected tropical diseases, Persistent diarrhoea, Côte d’Ivoire, Indonesia, Mali, Nepal

## Abstract

**Background:**

Diarrhoea still accounts for considerable mortality and morbidity worldwide. The highest burden is concentrated in tropical areas where populations lack access to clean water, adequate sanitation and hygiene. In contrast to acute diarrhoea (<14 days), the spectrum of pathogens that may give rise to persistent diarrhoea (≥14 days) and persistent abdominal pain is poorly understood. It is conceivable that pathogens causing neglected tropical diseases play a major role, but few studies investigated this issue. Clinical management and diagnostic work-up of persistent digestive disorders in the tropics therefore remain inadequate. Hence, important aspects regarding the pathogenesis, epidemiology, clinical symptomatology and treatment options for patients presenting with persistent diarrhoea and persistent abdominal pain should be investigated in multi-centric clinical studies.

**Methods/Design:**

This multi-country, prospective, non-experimental case–control study will assess persistent diarrhoea (≥14 days; in individuals aged ≥1 year) and persistent abdominal pain (≥14 days; in children/adolescents aged 1–18 years) in up to 2000 symptomatic patients and 2000 matched controls. Subjects from Côte d’Ivoire, Indonesia, Mali and Nepal will be clinically examined and interviewed using a detailed case report form. Additionally, each participant will provide a stool sample that will be examined using a suite of diagnostic methods (i.e., microscopic techniques, rapid diagnostic tests, stool culture and polymerase chain reaction) for the presence of bacterial and parasitic pathogens. Treatment will be offered to all infected participants and the clinical treatment response will be recorded. Data obtained will be utilised to develop patient-centred clinical algorithms that will be validated in primary health care centres in the four study countries in subsequent studies.

**Discussion:**

Our research will deepen the understanding of the importance of persistent diarrhoea and related digestive disorders in the tropics. A diversity of intestinal pathogens will be assessed for potential associations with persistent diarrhoea and persistent abdominal pain. Different diagnostic methods will be compared, clinical symptoms investigated and diagnosis-treatment algorithms developed for validation in selected primary health care centres. The findings from this study will improve differential diagnosis and evidence-based clinical management of digestive syndromes in the tropics.

**Trial registration:**

ClinicalTrials.gov; identifier: NCT02105714.

## Background

### Disease burden due to persistent digestive disorders in the tropics

**Fig. 1:**
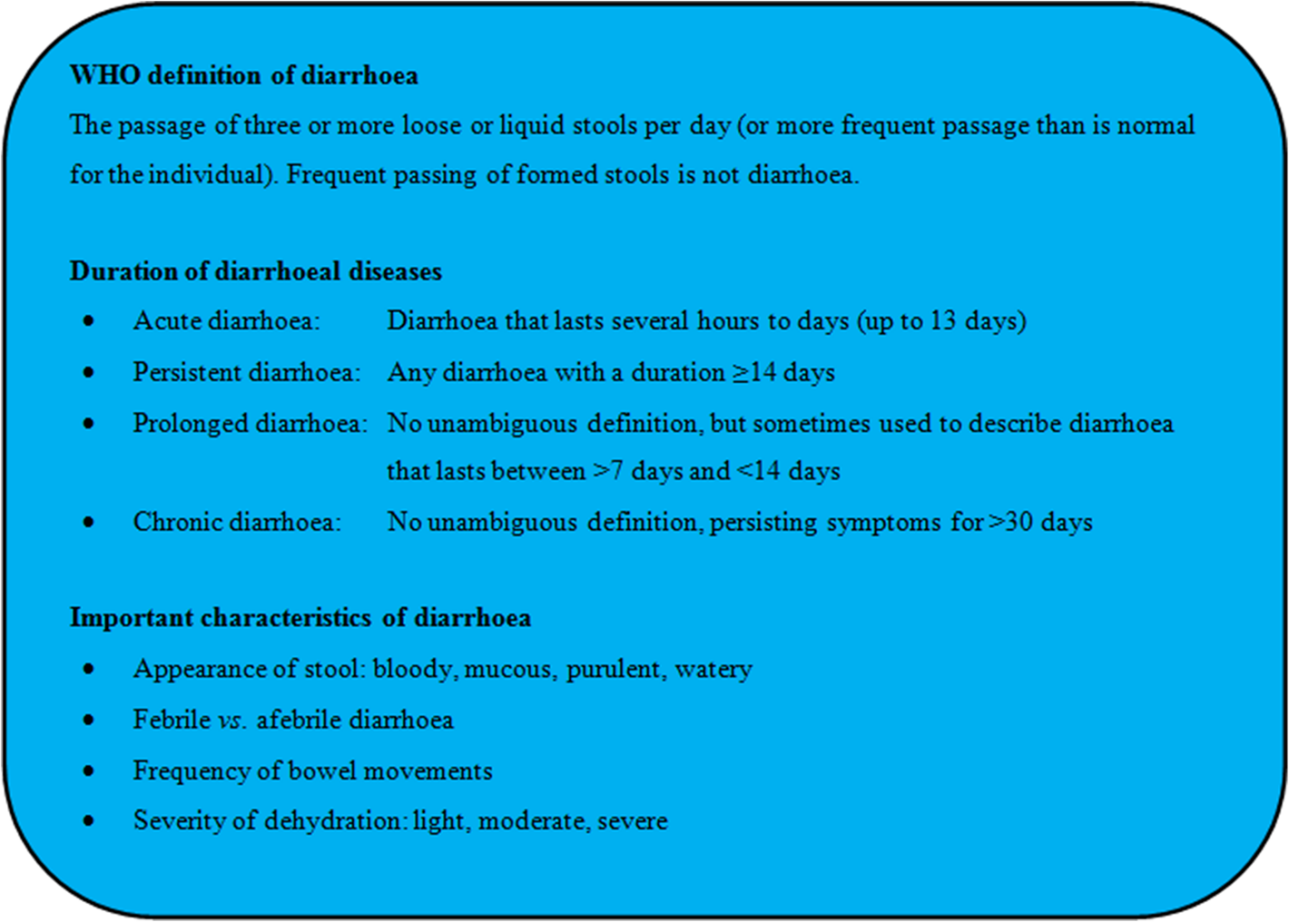
Synopsis of important definitions and characteristics of diarrhoeal diseases, based on recommendations put forth by the World Health Organization (WHO) and the Infectious Diseases Society of America (IDSA)

Diarrhoeal disorders are among the major communicable diseases worldwide, with a global burden second only to lower respiratory infections and greater than the burden of HIV/AIDS, malaria and tuberculosis [1]. Lack of access to clean water, improved sanitation and hygiene puts individuals in resource-constrained settings of tropical and subtropical countries at high risk of diarrhoea and other digestive disorders [2–4]. Indeed, severe disease manifestations and associated high mortality occur in children and immunocompromised individuals, particularly in low-income countries [5]. Diarrhoea is commonly defined as three or more loose stools per day and can be classified according to the total duration of disease (e.g., acute, prolonged, persistent and chronic), the severity (e.g., light, moderate and severe) and other characteristics (e.g., watery, mucous and bloody) [6]. Yet, some of these terms lack standardisation and are often used interchangeably, which renders comparison of different studies difficult. Figure 1 depicts definitions of diarrhoea, based on recommendations put forth by the World Health Organization (WHO) and expert guidelines [7].

While the epidemiology, aetiological pathogens and clinical management of acute diarrhoea have been extensively studied in both high-income countries and resource-constrained settings [8], far less attention has been addressed to persistent diarrhoea and other non-acute digestive disorders, such as persistent abdominal pain [9]. With regard to persistent diarrhoea, for example, it is conceived that parasitic infections (e.g., helminths and intestinal protozoa) are major pathogens to be considered. Bacterial and viral infections are thought to be of lesser importance, although the implication of bacteria in long-lasting diarrhoea is increasingly being recognised [10]. Current knowledge on parasitic pathogens giving rise to persistent diarrhoea mainly stems from experience gained in Western travel clinics and immunocompromised individuals, while there is a paucity of data from tropical areas [11, 12]. The few published studies focussed mainly on children, sample sizes were generally small and only a limited number of studies had an appropriate design (cohort studies, case–control studies) to properly investigate the true relationship between digestive symptoms and infections caused by specific pathogens [13]. In contrast, acute diarrhoeal diseases have been studied more intensively all over the world, and hence the aetiological spectrum is well characterised [8, 11]. In addition, the recent multi-country ‘Global Enteric Multicenter Study’ (GEMS) thoroughly investigated the causes of acute diarrhoea in infants and young children in developing countries [14, 15]. As for persistent diarrhoea, the authors of a systematic review published in 2009 concluded that further high quality studies are required to elaborate appropriate clinical guidelines [16]. The review brought to light that diarrhoeagenic *Escherichia coli* pathotypes were found in 30–40 % of children with persistent diarrhoea and intestinal protozoa in 15–20 % of them. Thus far, the potential contribution of helminths to this syndrome has not been studied, although some of these parasitic worms are classically considered as potential causes of persistent diarrhoea and persistent abdominal pain (e.g., *Schistosoma mansoni*, *Strongyloides stercoralis* and *Trichuris trichiura*) [17].

### Neglected tropical diseases and their contribution to persistent digestive disorders

The neglected tropical diseases (NTDs) comprise an evolving list of currently over 40 diseases that are caused by helminths (e.g., Schistosoma), intestinal protozoa (e.g., *Entamoeba histolytica*), bacteria (e.g., Shigella), viruses (e.g., dengue virus) and fungi (e.g., *Paracoccidioides braziliensis*) [18]. The global burden of NTDs accumulates to approximately 48 million disability-adjusted life years (DALYs) [19]. NTDs are closely linked to conditions of poverty which hinder social and economic development in endemic countries and negatively impact on people’s quality of life and wellbeing at many levels [20–22].

It is poorly understood to what extent NTDs may contribute to the clinical syndrome of persistent diarrhoea and persistent abdominal pain in resource-constrained settings. Long-lasting gastrointestinal complaints are a major reason for consultation of health centres or hospitals in the tropics, but few guidelines exist regarding appropriate clinical management. The problem is exacerbated by a lack of adequate diagnostic tools to guide treatment and control [23, 24]. If diagnostic techniques are available at all in low-income countries, they are usually not sensitive enough to detect NTDs with adequate accuracy (e.g., employment of unstained direct faecal smears for the microscopic diagnosis of intestinal parasites). Additionally, individuals with persistent digestive disorders are commonly managed as outpatients in underfinanced primary health care centres in rural areas, where health system resources are even weaker than in hospital settings [25–28]. Due to the lack of adequate diagnostics and the uncertain role NTDs play as causative agents of persistent digestive disorders, clinicians may frequently assume a bacterial infection and prescribe antibiotic treatment without detailed diagnostic work-up. If symptoms persist or deteriorate despite anti-infective therapy, empirical treatment with one or several antiparasitic drugs may be added. However, it is unknown whether such an approach is medically appropriate, whether adequate clinical cure rates can be achieved and whether this strategy is cost-effective [29].

### NIDIAG: developing diagnosis-treatment algorithms for neglected clinical syndromes

NIDIAG is an international collaborative research consortium that aims at improved clinical management of common clinical syndromes in the tropics (http://www.nidiag.org). This 5-year project is funded by the European Commission (EC), Framework Programme 7. NIDIAG aims to develop improved, patient-centred approaches to be applied in primary health care centres of resource-constrained settings. Three clinical syndromes are being investigated: (i) persistent digestive disorders [9]; (ii) persistent fever [30, 31]; and (iii) neurological disorders [32]. Particular emphasis is placed on the contribution of NTDs to each syndrome.

The current study protocol focuses on persistent digestive disorders, which are defined as diarrhoea (≥14 days) in individuals aged ≥1 year and/or abdominal pain (≥14 days) in children and adolescents aged 1–18 years. Adults presenting only with abdominal pain will not be studied because a considerable proportion of persistent digestive symptoms is due to non-infectious causes and beyond the scope of this study [33]. However, children presenting with persistent abdominal pain will be included, even in the absence of diarrhoea. Indeed, it is commonly assumed among tropical clinicians that long-lasting abdominal pain is associated with parasitic and other intestinal infections in this age group, but further clinical evidence is warranted [34].

### Goal, aims and objectives

The overarching goal of this study is to develop clinical algorithms that provide an evidence-based syndromic approach to NTDs at the primary health care level and shall lead to better diagnosis and management of patients with persistent digestive disorders in resource-constrained settings. To this end, we will pursue a multi-centric, prospective case–control study in Côte d’Ivoire, Indonesia, Mali and Nepal. Patients (≥1 year old) presenting with persistent diarrhoea (≥14 days) and/or children and adolescents (aged 1–18 years) with persistent abdominal pain (≥14 days) will be enrolled. Insights gained during the study will help to develop and, in a second study phase, to validate readily applicable diagnostic algorithms that shall facilitate the clinical decision-making and management of patients presenting with persistent diarrhoea and abdominal pain. The following aims and specific objectives are related to this goal.

#### Aims

1. To improve the quality of clinical care for persistent diarrhoea and persistent abdominal pain through the development of evidence-based diagnosis-treatment algorithms for use in primary health care centres.
2. To identify the major NTDs and other infectious agents (i.e., bacteria and parasites) that give rise to persistent digestive disorders and to assess their relative contribution to this clinical syndrome.
3. To compare different diagnostic methods and to assess their diagnostic accuracy, including clinical features, conventional laboratory techniques, rapid diagnostic tests (RDTs) and molecular assays for the diagnosis of selected pathogens.
4. To assess the clinical response to commonly employed empirical treatment options for persistent digestive disorders.

#### Specific objectives and activities

1. To examine stool samples from symptomatic patients presenting with persistent diarrhoea and children/adolescents with persistent abdominal pain with a range of standardised, quality-controlled laboratory techniques (e.g., microscopic techniques, RDTs and stool culture) for detection of parasites and pathogenic bacteria.
2. To examine stool samples from matched healthy controls without gastrointestinal complaints with the same suite of diagnostic tests and approaches.
3. To retrospectively analyse a (sub-)sample of stool specimens using multiplex polymerase chain reaction (PCR) assays in reference laboratories. PCR tests will be used to assess the diagnostic accuracy of conventional tests, as well as to obtain data on additional pathogens.
4. To assess the relative importance of the pathogens detected in symptomatic individuals, as compared to healthy controls.
5. To perform a standardised medical examination on all participants and to accurately document clinical signs and symptoms, risk factors, clinical management and response to treatment in a case report form (CRF).
6. To calculate the predictive values of clinical and laboratory data to provide evidence-based data for the development of a diagnostic algorithm.
7. To develop and validate one or several diagnosis-treatment algorithms for the management of persistent digestive disorders in primary health care settings of resource-limited countries.
8. To assess the cost-effectiveness of different approaches for the diagnosis of selected NTDs in patients presenting with persistent digestive disorders.

## Methods/Design

### Study area

For this study, four low- or middle-income countries, two in West Africa (Côte d’Ivoire and Mali) and two in Asia (Indonesia and Nepal), were selected as study sites to cover a broad geographic range, diverse pathogen profiles and different health systems. Prior to the selection of the study sites, an extensive review of existing epidemiological data on diarrhoeal diseases and the targeted NTDs in the study countries was conducted. An assessment of currently employed diagnosis-treatment practices and ongoing control activities in each country confirmed that all four countries are endemic for the main target NTDs/pathogens under investigation. In Côte d’Ivoire and Mali, currently no national guidelines or algorithms exist for the diagnosis and treatment of persistent diarrhoea or persistent abdominal pain. In Nepal, some paediatric guidelines exist, but no standardised approach towards persistent diarrhoea in adults has been developed thus far. Indonesia is the only country where, since 2011, a national programme for the management of diarrhoeal diseases is in place. All study countries have national programmes for control of NTDs, but there is considerable variation with regard to control approaches and the spectrum of NTDs covered, varying from strategies that target exclusively intestinal helminths (e.g., in Nepal) to multidimensional integrated NTD control programmes (e.g., in Mali) [35].

Our research will be conducted in peripheral health centres and referral level hospitals in order to capture a large variety of clinical presentations of the digestive syndrome. The study sites were selected on the basis of the following criteria: (i) location in remote rural areas or deprived urban settings; (ii) existing referral links between primary health care centre and district hospital (e.g., for diagnosis and treatment of complicated or severe conditions); (iii) adequate patient care in the referral hospitals; (iv) persistent digestive disorders being a relevant and frequently occurring clinical syndrome, as judged by the local physicians and nurses; (v) available laboratory infrastructure and staff capacity to perform standard first-line tests and to process field samples for external work-up; and (vi) key staff trained in good clinical practice (GCP) and good clinical laboratory practice (GCLP).

**Fig. 2:**
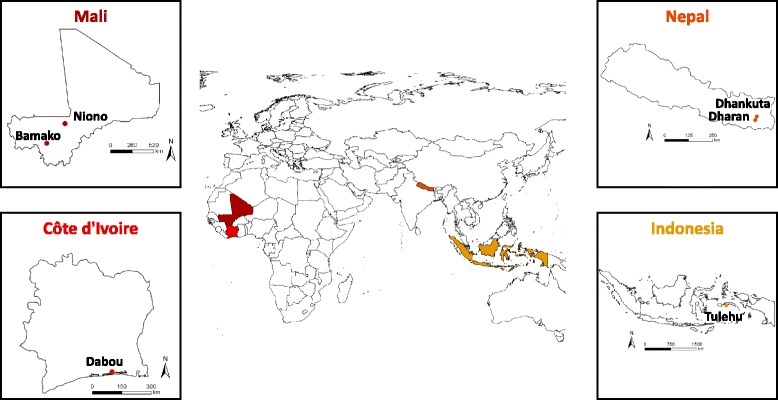
World map and country-specific maps of Côte d’Ivoire, Indonesia, Mali and Nepal, indicating the sites of patient recruitment for the NIDIAG study on persistent digestive disorders

Country-specific assessments were carried out during the site selection procedure. The following seven study centres were selected in the four study countries (Fig. 2):

- Côte d’Ivoire
  - Hôpital Méthodiste de Dabou, a regional reference hospital based in Dabou, approximately 30 km west of Abidjan, the country’s economic capital.
- Indonesia
  - Tulehu Hospital, based in Tulehu in Maluku province, approximately 100 km northeast of Ambon City, the province’s capital; and
  - Tulehu Health Centre, also situated in Maluku province in close proximity (approximately 1 km) to the Tulehu Hospital.
- Mali
  - Niono District Reference Health Centre, based in Niono, approximately 300 km northeast of the country’s capital Bamako; and
  - Institut National de Recherche en Santé Publique (INRSP) in Bamako.
- Nepal
  - B P Koirala Institute of Health Sciences, situated in Dharan, approximately 350 km southeast of the country’s capital Kathmandu; and
  - Dhankuta District Hospital in Dhankuta, approximately 50 km north of Dharan.

### Study design

Our investigation is designed as a prospective, non-experimental case–control study to determine the relative importance of NTDs in patients who present with persistent digestive disorders. The case–control design was adopted because of the observation that multiple digestive pathogens may be found even in asymptomatic individuals, and hence these may rather represent ‘innocent bystanders’ that do not play a causal role in the aetiology of the persistent symptomatology [36, 37]. We believe that the inclusion of a control group will provide discriminative data on the distribution of pathogens and might be useful to enhance interpretation of causal associations [38, 39].

### Study participants

The inclusion criteria for cases are (i) all individuals (aged ≥1 year) presenting with persistent diarrhoea (≥14 days); and/or (ii) children/adolescents (aged 1–18 years) with persistent abdominal pain (≥14 days). For persistent diarrhoea, the WHO definitions are used (see also Fig. 1). WHO defines diarrhoea as the passing of three or more loose stools within 24 h. A new episode of diarrhoea can occur after two full days without diarrhoea. Episodes of diarrhoea lasting for 14 days and longer are defined as persistent [6]. For persistent abdominal pain, no official WHO definition exists. Here, we define persistent abdominal pain as localised or diffuse abdominal pain lasting for at least 14 days (possibly with intermittence/recurrence). To each enrolled patient, one control without any gastrointestinal complaints will be matched by age group, sex and geographical location of residence. These may be patients who present to the same hospital or outpatient facility as the case, but with non-related complaints (e.g., with ophthalmological diseases or consulting for vaccination or ‘routine’ check-up). If the patient was referred to the hospital by one of the peripheral health facilities in the catchment area, the control will be (actively) selected through the same or a nearby peripheral health facility.

Patients and controls presenting with the following characteristics will be excluded: (i) unwilling or unable to give informed consent; (ii) unable in the study physician’s opinion to comply with the study requirements; (iii) presenting with clinical jaundice (as assessed by direct observation of the conjunctivae); (iv) already participating in other ongoing diagnostic studies and/or clinical trials; and (v) in need of immediate intensive or surgical care (including severely malnourished children).

### Sample size

The lack of available data on the frequency of persistent digestive disorders in resource-constrained settings and the diversity of implicated infectious pathogens renders sample size calculations difficult [40]. A systematic review on pathogens associated with persistent diarrhoea in children in low- and middle-income countries reported prevalences of about 10 % for *Giardia intestinalis*, 5–10 % for *Campylobacter* spp. and around 5 % for *Cryptosporidium* spp. [16]. Prior data from Côte d’Ivoire [37, 41] indicate that the probability of infection with target pathogens such as *S. stercoralis* is around 10-15 % in symptomatic cases and 5–10 % in healthy controls. If the true probability of infection among cases is 10 %, 435 cases and 435 controls need to be included to be able to reject the null hypothesis that the infection rates for case and controls are identical with a probability (power) of 0.8 (2-sided alpha = 0.05). To compensate for loss to follow-up of 10 %, we aim at enrolling a total of 500 cases and 500 controls in each of the four study countries. It should be noted, however, that no robust information exist to date, and hence most data and resulting calculations remain somewhat speculative. As most reported prevalences of several important target NTDs (e.g., hookworm and *G. intestinalis*) are in the same range as described above, we adhered to these assumptions, and hence, we aim for a total of 2000 patients and 2000 matched controls.

### Patient recruitment and clinical management

**Fig. 3:**
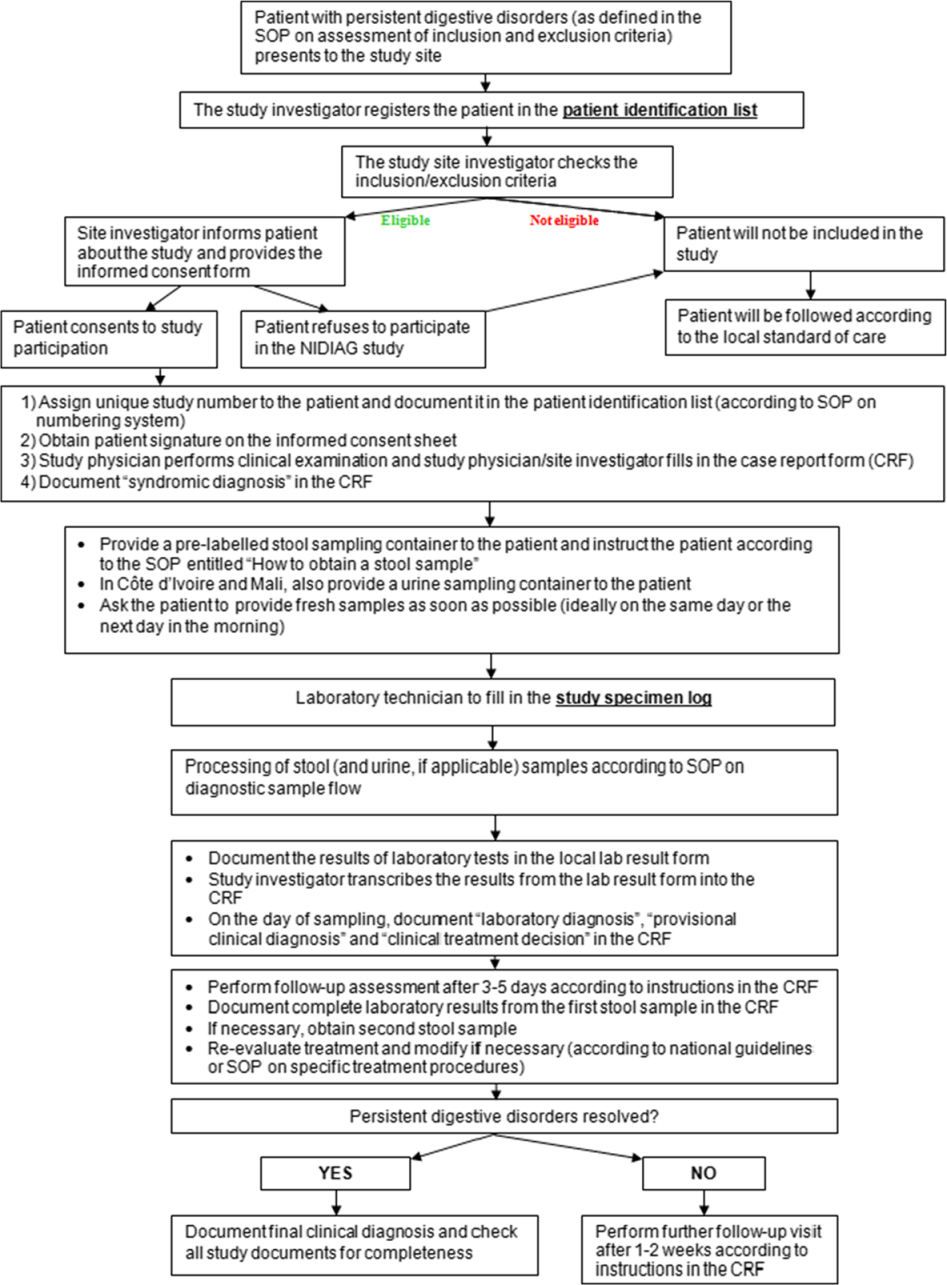
Patient flow of the NIDIAG study on persistent digestive disorders, as outlined in a specific standardised operating procedure (SOP)

The recruitment strategy will be adapted to the local setting, taking into consideration the local norms, culture and health care system organisation. Before the start of the study, appropriate context-adapted mechanisms will be set up to inform the community’s representatives in the catchment area of the respective study centres about the study, its objectives, the implications for the community and potential future impacts. All procedures during the study will be guided by an extensive set of specifically developed standardised operating procedures (SOPs). Figure 3 shows the patient recruitment and study flow according to the respective SOPs. Whenever a patient presents to one of the study centres with self-reported persistent digestive disorders (≥14 days), the care provider will immediately contact the study investigator. The study investigator will assess inclusion/exclusion criteria by medical history taking, review of existing medical documents (e.g., results of laboratory tests, note from referring physician) and a rapid clinical assessment. Patients who meet the inclusion criteria will be invited to participate in the trial. Once informed consent has been obtained, the selected individuals will undergo a full clinical assessment and will be asked to provide one fresh stool sample. In the two West African countries, an additional urine sample will be obtained for diagnostic work-up. Based on the initial assessment, the study physician will document a syndromic diagnosis (e.g., suspected parasitic intestinal infection) as well as any therapeutic decision (e.g., initiation of empirical treatment). Of note, all decisions will be documented, but the current practices of the clinician will not be influenced (non-interventional trial). All patients will be followed and a systematic second visit for in-depth evaluation will be conducted 3–5 days after the first visit. By the time of the second visit, all microbiological laboratory results will be available and the treatment may be adapted in case of a specific detected pathogen or an unsatisfactory clinical evolution. Patients with persistent symptoms will be asked to provide another stool sample for detailed diagnostic work-up. If need be, a patient might be assessed during a third visit that is performed one to two weeks after the second visit. In case of clinical complaints being still present, a third stool sample may be tested and treatment might be modified again. Additional visits and further laboratory work-up will be left to the discretion of the care provider. Each time, new or persistent clinical symptoms and signs as well as all treatment decisions will be recorded. The final diagnosis for each patient will be based on the combination of laboratory tests, clinical response to treatment and additional investigations requested by the treating clinician (e.g., abdominal ultrasound examination).

Individuals without any gastrointestinal complaints will be invited to participate as controls in this trial. After informed consent has been obtained, the diagnostic procedures applied to the controls are identical as for the cases.

### Diagnostic tests and laboratory procedures

All laboratory activities will be carried out according to the specific SOPs. The diagnostic tests to be employed were selected after a systematic review of the peer-reviewed literature and standard textbooks, supplemented with expert opinion [9]. Four different classes of tests will be employed during the study, namely (i) stool microscopy and stool concentration techniques for the diagnosis of intestinal protozoa and helminths (on site; all study countries); (ii) a stool-based RDT for the diagnosis of *Cryptosporidium* spp. and *G. intestinalis* (on site; all study countries) and a urine-based RDT for the detection of *S. mansoni* (on site; only in Côte d’Ivoire and Mali); (iii) bacteriological stool culture for the diagnosis of selected enteric bacteria in patients presenting with persistent diarrhoea (in national reference laboratories; all study countries except Indonesia, where the capacity and available laboratory equipment to perform bacteriological stool cultures in the selected centre did not meet the required reference standards); and (iv) PCR assays for the detection of parasitic and, possibly, additional bacterial and viral pathogens (in European reference laboratories; ethanol-fixed stool samples from all study countries). Table 1 provides an overview of the applied tests and the pathogens that can be detected by the individual techniques. Most of the test results will be available within 3 days after provision of the stool sample so that targeted treatment of the detected pathogen can be offered to the patient, based on national guidelines and evidence-based treatment recommendations. However, it is important to note that the PCR tests will only be conducted several weeks or months after stool sampling, and hence, these test results will not be available in a timely manner to guide clinical patient management.

**Table 1:** Diagnostic tests to be employed on stool (and urine) samples from patients with persistent digestive disorders and healthy controls in Côte d’Ivoire, Indonesia, Mali and Nepal during the NIDIAG study. Note: except for the rapid diagnostic test (RDT) for *Schistosoma mansoni* which uses urine, all tests are performed on stool samples

Diagnostic test	Targeted pathogens	Reference(s)
Microscopy		
Direct faecal smear	Helminths, intestinal protozoa	[55]
Kato-Katz thick smear	Helminths	[56]
Acid-fast staining	*Cryptosporidium* spp., *Cyclospora cayetanensis*, *Cystoisospora belli*	[57]
Baermann funnel concentration technique	*Strongyloides stercoralis*, hookworm	[41, 58]
Formalin-ether concentration technique	Helminths, intestinal protozoa	[59, 60]
Mini-FLOTAC	Helminths	[61, 62, 63]
Culture		
Bacteriological stool culture	*Salmonella* spp., *Shigella* spp., *Campylobacter* spp., *Yersinia* spp.	[9]
Koga agar culture	*Strongyloides stercoralis*, hookworm	[64–67]
Rapid diagnostic tests (RDTs)		
Crypto/Giardia DuoStrip	*Cryptosporidium* spp., *Giardia intestinalis*	[37]
Circulating cathodic antigen (CCA)^a^	*Schistosoma mansoni*	[68–72]
Molecular post-hoc testing on ethanol-fixed stool samples		
Multiplex PCR	Helminths, intestinal protozoa (all samples); diarrhoeagenic bacteria and viruses (selected sub-sample)	[73–76]

^a^This test will only be employed in Côte d’Ivoire and Mali, because *S. mansoni* does not occur in Indonesia and Nepal

### Laboratory quality control and monitoring

We will adhere to rigorous quality control procedures for all laboratory investigations specified in Table 1. A ‘quality manager’ will be designated in each study site to actively monitor the laboratory activities throughout the study. This person will verify the storage conditions and accountability of the reagents used for the studies. It will be carefully checked that samples are collected, transferred, processed, analysed and stored according to SOPs. Moreover, 5–10 % of all processed stool samples will be re-read under a microscope by experienced laboratory technicians. A photo of each RDT result will be taken at the time of reading, and subsequently documented. The photos and the recorded results will be checked for consistency by the quality manager. Additionally, external monitoring will be conducted at regular intervals, and according to GCLP standards. These measures will ensure quality and reproducibility of all laboratory results within and across study countries.

### Economic evaluation

The cost and cost-effectiveness of the different diagnostic laboratory tests and diagnosis-treatment algorithms will be assessed and compared to existing practice. The economic evaluation will take the perspective of the public health care system, focussing on practice and implementation at the primary care level. The costing of the baseline and the diagnosis-treatment algorithm, including all diagnostic tests used in the study, will be done using a combination of top-down and bottom-up costing methodologies [42]. Information will be collected on recurrent items (e.g., staff, supplies and reagents) and capital costs (e.g., building, equipment and furniture). For the estimation of the unit costs of diagnostic tests, information on resource usage will be collected through time-and-motion studies. Resources will be valued at their opportunity cost, including the value of donated or subsidised items (i.e., economic costing). Capital items will be annualised over their expected life span.

### Data collection

All clinical and laboratory data will be recorded in a specifically designed and pre-tested CRF. Two distinct CRFs were developed; one for patients and one for controls. The study investigator/clinician will complete one CRF for each study participant to document demographic data (e.g., age, sex and residency), clinical data (e.g., medical history, including data on previous exposure and risk behaviour, current symptoms, physical examination, new or persistent clinical symptoms and signs, clinical evolution, final clinical outcome), laboratory data (e.g., clinical sampling, test results for each sample, laboratory evolution), diagnosis (e.g., syndromic diagnosis, working and final diagnosis based on available clinical and laboratory results) and treatment decisions (e.g., abstention or initiation of empirical or targeted treatment, additional treatments, treatment modifications and treatment response). All treatments are recorded in a separate document (medication form). Any additional clinical or laboratory investigations as well as unscheduled visits will be documented. The consistency and quality of data collection will be checked regularly by an internal quality control manager and during the recruitment phase via external monitor visits.

### Data management and storage of data

Data management at the respective study centres will be carried out under the guidance and supervision of a central data manager. The local data manager will be responsible for supervising data entry and local data management. Completed CRFs will be submitted to the local data management, where data of the CRF will be recorded into the study database. After data cleaning and database lock, the database will be shared with the collaborating institutes. All data entry and management will be done by trained study personnel. As required by international guidelines and national regulations, the study file, signed informed consent sheets, source documents, copy of the CRF and subject identification codes will be retained for at least 2 years (or for the time period required by national legislation) at each study site (ICH-GCP 4.9.5) to allow for audits and inspections even after the study completion. The study investigators are responsible for reporting a patient’s and control’s personal details and identification number in a participant identification list. To ensure confidentiality, this list will be kept in a separate, locked cupboard together with the signed informed consent forms and only the study investigators and clinical study staff will have access to it.

All laboratory specimens, including preserved samples, and all subject-related reports, forms and study data will be identified by a coded number. Subject names will not be used. All local databases will be secured with password-protected access systems. A list of authorised personnel to access the databases throughout the study will be held. Regular backups of the database at the study sites will be performed and shared with the central data manager. Computers and other study hardware (e.g., external memory to store backups) will be used only for study purposes and kept in locked cupboards and/or rooms. Any personal study information will not be released to anybody outside the medical team, except to competent authorities for independent monitoring, auditing and inspection. Throughout the study, strict confidentiality, quality, and security of the subjects’ data will be pursued.

### Data analysis

Statistical analyses will be carried out with STATA version 12 (StataCorp LP; College Station, USA) or SPSS Statistics version 22 (IBM Corporation; Armonk, USA). The prevalence and, in case of parasitic infections, the intensity of infection with targeted NTDs will be utilised as primary outcome measure. Logistic regression models will be employed to estimate associations of putative pathogens with persistent digestive disorders. The aetiological fraction for matched case–control studies will be calculated to estimate the fraction of cases with digestive disorders due to a specific pathogen. The sensitivity of clinical and laboratory predictors and of RDTs will be calculated as the proportion of patients with a given disease (“confirmed” diagnosis only) who present with a clinical feature or show a positive test result. The specificity of clinical and laboratory predictors and of RDTs will be calculated as the proportion of individuals without the disease (“confirmed” diagnosis only) who do not present with this feature or show a negative test result. Positive and negative likelihood ratio of clinical and laboratory predictors and of RDTs will be calculated using standard formulas. The positive likelihood ratio (LR+) will be obtained by dividing the sensitivity of individual predictor by 1 minus the specificity of the predictor. The negative likelihood ratio (LR-) will be obtained by dividing 1 minus the sensitivity of the predictor by its specificity. The positive predictive value (PPV) of clinical and laboratory predictors and of RDTs as well as their combination will be calculated according to Bayes’ formula as the proportion of patients with a clinical feature or a positive test result who have the disease. The negative predictive value (NPV) of clinical and laboratory predictors and of RDTs as well as their combination will be calculated as the proportion of individuals without this feature or with a negative test result who do not have the disease.

### Ethical approval

The study protocol was discussed, reviewed and modified by several experts within the NIDIAG research consortium and its independent scientific and ethical board. It was approved by the institutional review boards (IRBs) at the Institute of Tropical Medicine (ITM) in Antwerp, Belgium and the Swiss Tropical and Public Health Institute (Swiss TPH) in Basel, Switzerland prior to external review. Country-specific approvals were subsequently granted by the followings ethics committees: (i) University of Antwerp in Belgium (12 August 2013); (ii) Gadjah Mada University in Indonesia (21 November 2013); (iii) ‘Ethikkommission beider Basel’ (EKBB) in Switzerland (22 November 2013); (iv) ‘Institut National de Recherche en Santé Publique’ in Mali (28 November 2013); (v) Ministry of Health in Côte d’Ivoire (28 November 2013); and (vi) ‘Nepal Health Research Council’ (NHRC) in Nepal (29 January 2014). The trial is registered on ClinicalTrials.gov (identifier: NCT02105714). Should any ethical issues arise during the conduct of the study, these will be promptly discussed within the NIDIAG ethical board and referred to the concerned IRBs.

### Policy and dissemination strategy

NIDIAG aims to improve quality of clinical care for three common syndromes in the tropics and will provide specific efforts to translate evidence into policy. Hence, policy makers will be involved in the project from an early stage and will be consulted during the development of the following decision support tools: (i) development and validation of algorithms for the clinical management of persistent digestive disorders; (ii) cost estimates and cost-effectiveness analyses of integrated algorithms and diagnostic platforms compared to single-disease oriented laboratory tests; and (iii) analysis of acceptability and appropriateness of innovative diagnosis-treatment tools for application in the primary health care settings. A comprehensive translation-to-policy strategy will be put in place and insights gained through the NIDIAG study will be widely disseminated among patient groups and participating communities.

## Discussion

Digestive disorders are among the major clinical syndromes that characterise a host of NTDs in Africa, Asia, Latin America and elsewhere. The morbidity due to diarrhoeal diseases in low- and middle-income countries continues to be considerable. While the aetiological spectrum of acute diarrhoea has been widely investigated, the major pathogens giving rise to persistent diarrhoea and other non-acute digestive disorders remain to be elucidated [9, 16].

There is a pressing need to assess frequently encountered clinical syndromes in resource-constrained settings from a patient-centred perspective, rather than relying on single disease-oriented approaches. Indeed, a recent study in Tanzania investigated the causes of fever in children and brought to light that viral infections predominate and are considerably more common than parasitic diseases (e.g., malaria) and bacterial infections (e.g., bacterial pneumonia) [43]. In contrast to this finding, clinicians commonly prescribe antimalarial and antibacterial medication in the absence of diagnostic testing facilities, which in turn leads to overtreatment with potential negative consequences (e.g., development of resistance and drug toxicity). It is conceivable that similar misconceptions and mismanagement apply to other clinical syndromes, including persistent diarrhoea and persistent abdominal pain, which is the focus of the current study protocol. It has been postulated that an improved syndromic approach to highly prevalent complaints such as persistent diarrhoea, persistent abdominal pain or long-lasting fever will require (i) a deeper understanding of the aetiology and epidemiology of the target diseases; (ii) the availability of readily applicable RDTs in the most affected areas; and (iii) the development of evidence-based algorithms for the management of these syndromes [30]. NIDIAG aims to address these issues for three clinical syndromes: the persistent digestive disorders discussed here, along with neurological disorders and persistent fever.

Several considerations underscore the need for a concerted, multi-centric assessment of persistent diarrhoea and persistent abdominal pain in resource-constrained settings, with particular emphasis on the primary health care level. First, digestive syndromes are associated with many different NTDs, particularly helminth and intestinal protozoa infections [44], but the magnitude of their contribution to persistent digestive disorders is largely unknown. A single disease perspective will not be able to determine the relative importance of NTDs in patients who present with persistent diarrhoea and persistent abdominal pain; hence a syndromic and patient-centred approach seems more promising. Second, for some of the NTDs that may give rise to digestive disorders, public health programmes to control these infections have been implemented [45]. For instance, the control of several helminth NTDs currently relies on preventive chemotherapy; that is the regular, large-scale administration of one or several orally available drugs with the aim of reducing the parasite load and thus preventing the development of severe disease [46, 47]. These strategies offer opportunities for a rapid impact at low cost in resource-constrained settings, especially in areas where the targeted diseases are highly prevalent. However, the long-term sustainability of these programmes has been questioned [48, 49]. Recently, public health specialists put particular emphasis on integrated control approaches that go beyond preventive chemotherapy [50, 51]. The disease-specific focus of many NTD control programmes has led to fragmentation and gaps in patient management. At the primary health care level, however, nurses and physicians do not deal with a single disease, but with patients presenting with a wide spectrum of complaints. Among these clinical syndromes, they have to recognise the specific NTDs and discriminate these from a constellation of other illnesses. Hence, there is a need for evidence-based guidelines on the management of a specific clinical syndrome and the contribution of NTDs rather than single programmes targeting one of the aetiological agents. There is growing consensus that well-functioning health systems are required to achieve sustainable NTD control and improved diagnosis-treatment algorithms are key to this [52, 53]. Third, as control of selected NTDs is moving towards elimination in selected settings, it will be important to conduct studies in the primary health care settings to allow for an unbiased detection and surveillance of these infections. Such an assessment can only be achieved on a large scale by the first-line caregivers and available evidence-based algorithms for diagnostic work-up would provide a useful tool for patient management.

Taken together, the NIDIAG digestive study offers an integrated syndromic approach to NTD-related clinical syndromes, focussing on digestive disorders defined as persistent diarrhoea (≥14 days of diarrhoea in all individuals aged ≥1 year) and persistent abdominal pain (≥14 days in children and adolescents aged 1–18 years). The overarching goal is the elaboration of evidence-based diagnosis-treatment algorithms centred on patients in resource-constrained settings, where available data are scant and patient management is often driven by ‘empirical evidence’ and local beliefs. Furthermore, this study will help to assess the contribution of NTDs to persistent digestive disorders and, subsequently, develop and evaluate sustainable and cost-effective control programmes for these infections. Additionally, the NIDIAG digestive study will optimise the use of existing diagnostic tests and advance the development of new methods, which must be simple to use, affordable for application in low-income countries and able to detect a broad spectrum of intestinal pathogens with high accuracy [54]. Finally, the insights gained during this multi-country prospective study will also help to re-estimate the burden of persistent digestive disorders and may influence future public health recommendations and health policy planning.
