# Erratum to: Diagnosis of neglected tropical diseases among patients with persistent digestive disorders (diarrhoea and/or abdominal pain ≥14 days): a multi-country, prospective, non-experimental case–control study

**DOI:** 10.1186/s12879-015-1160-0

**Published:** 2015-11-04

**Authors:** Katja Polman, Sören L. Becker, Emilie Alirol, Nisha K. Bhatta, Narayan R. Bhattarai, Emmanuel Bottieau, Martin W. Bratschi, Sakib Burza, Jean T. Coulibaly, Mama N. Doumbia, Ninon S. Horié, Jan Jacobs, Basudha Khanal, Aly Landouré, Yodi Mahendradhata, Filip Meheus, Pascal Mertens, Fransiska Meyanti, Elsa H. Murhandarwati, Eliézer K. N’Goran, Rosanna W. Peeling, Raffaella Ravinetto, Suman Rijal, Moussa Sacko, Rénion Saye, Pierre H. H. Schneeberger, Céline Schurmans, Kigbafori D. Silué, Jarir A. Thobari, Mamadou S. Traoré, Lisette van Lieshout, Harry van Loen, Kristien Verdonck, Lutz von Müller, Cédric P. Yansouni, Joel A. Yao, Patrick K. Yao, Peiling Yap, Marleen Boelaert, François Chappuis, Jürg Utzinger

**Affiliations:** Department of Biomedical Sciences, Institute of Tropical Medicine, Antwerp, Belgium; Department of Epidemiology and Public Health, Swiss Tropical and Public Health Institute, Basel, Switzerland; University of Basel, Basel, Switzerland; Institute of Medical Microbiology and Hygiene, Saarland University, Homburg, Germany; Institute of Medical Microbiology and Hygiene, Saarland University, Saar, Germany; Division of Tropical and Humanitarian Medicine, Geneva University Hospitals, Geneva, Switzerland; Department of Paediatrics and Adolescent Medicine, B P Koirala Institute of Health Sciences, Dharan, Nepal; Department of Microbiology, B P Koirala Institute of Health Sciences, Dharan, Nepal; Department of Clinical Sciences, Institute of Tropical Medicine, Antwerp, Belgium; London School of Hygiene and Tropical Medicine, London, UK; Unité de Formation et de Recherche Biosciences, Université Félix Houphouët-Boigny, Abidjan, Côte d’Ivoire; Département Environnement et Santé, Centre Suisse de Recherches Scientifiques en Côte d’Ivoire, Abidjan, Côte d’Ivoire; Institut National de Recherche en Santé Publique, Bamako, Mali; Centre for Tropical Medicine, Faculty of Medicine, Gadjah Mada University, Yogyakarta, Indonesia; University of Cape Town, Cape Town, South Africa; Coris BioConcept, Gembloux, Belgium; Department of Pharmaceutical and Pharmacological Sciences, KU Leuven, Leuven, Belgium; Department of Internal Medicine, B P Koirala Institute of Health Sciences, Dharan, Nepal; Department of Epidemiology and Molecular Diagnostics, Agroscope Changins-Wädenswil ACW, Wädenswil, Switzerland; Department of Virology, Spiez Laboratory, Federal Office for Civil Protection, Spiez, Switzerland; Department of Parasitology, Leiden University Medical Center, Leiden, The Netherlands; Department of Public Health, Institute of Tropical Medicine, Antwerp, Belgium; Divisions of Infectious Diseases and Medical Microbiology, J.D. MacLean Centre for Tropical Diseases, McGill University Health Centre, Montreal, Canada

Unfortunately the original article [1] contained a typo in the title. Instead of “Diagnosis of neglected tropical diseases among patients with persistent digestive disorders (diarrhoea and/or abdominal pain ≥14 days): a multi-country, prospective, non-experimental case–control study” the articles was accidentally called “Diagnosis of neglected tropical diseases among patients with persistent digestive disorders (diarrhoea and/or abdominal pain ≥14 days): Pierrea multi-country, prospective, non-experimental case–control study”. This has now been updated in the original article and corrected with this erratum.
